# Comparative genomics of molybdenum utilization in prokaryotes and eukaryotes

**DOI:** 10.1186/s12864-018-5068-0

**Published:** 2018-09-19

**Authors:** Ting Peng, Yinzhen Xu, Yan Zhang

**Affiliations:** 1 Shenzhen Key Laboratory of Marine Bioresources and Ecology, College of Life Sciences and Oceanography, Shenzhen University, Guangdong Province, Shenzhen, 518060 China; 2 Shanghai Institute of Nutrition and Health, Shanghai Institutes for Biological Sciences, University of Chinese Academy of Sciences, Chinese Academy of Sciences, Shanghai, 200031 China

**Keywords:** Molybdenum, Moco, Molybdoprotein, Comparative genomics, Evolution

## Abstract

**Background:**

Molybdenum (Mo) is an essential micronutrient for almost all biological systems, which holds key positions in several enzymes involved in carbon, nitrogen and sulfur metabolism. In general, this transition metal needs to be coordinated to a unique pterin, thus forming a prosthetic group named molybdenum cofactor (Moco) at the catalytic sites of molybdoenzymes. The biochemical functions of many molybdoenzymes have been characterized; however, comprehensive analyses of the evolution of Mo metabolism and molybdoproteomes are quite limited.

**Results:**

In this study, we analyzed almost 5900 sequenced organisms to examine the occurrence of the Mo utilization trait at the levels of Mo transport system, Moco biosynthetic pathway and molybdoproteins in all three domains of life. A global map of Moco biosynthesis and molybdoproteins has been generated, which shows the most detailed understanding of Mo utilization in prokaryotes and eukaryotes so far. Our results revealed that most prokaryotes and all higher eukaryotes utilize Mo whereas many unicellular eukaryotes such as parasites and most yeasts lost the ability to use this metal. By characterizing the molybdoproteomes of all organisms, we found many new molybdoprotein-rich species, especially in bacteria. A variety of new domain fusions were detected for different molybdoprotein families, suggesting the presence of novel proteins that are functionally linked to molybdoproteins or Moco biosynthesis. Moreover, horizontal gene transfer event involving both the Moco biosynthetic pathway and molybdoproteins was identified. Finally, analysis of the relationship between environmental factors and Mo utilization showed new evolutionary trends of the Mo utilization trait.

**Conclusions:**

Our data provide new insights into the evolutionary history of Mo utilization in nature.

**Electronic supplementary material:**

The online version of this article (10.1186/s12864-018-5068-0) contains supplementary material, which is available to authorized users.

## Background

The transition element molybdenum (Mo) is of essential importance for a number of molybdoproteins in almost all living organisms from bacteria to humans, where it functions as a catalytic component of these enzymes [1, 2]. Except for the iron (Fe)-Mo cofactor detected in nitrogenase, Mo is complexed by a specific pyranopterin moiety (referred to as molybdopterin), thereby generating the molybdenum cofactor (Moco) in all molybdoproteins [1–4]. In some prokaryotes such as (hyper)thermophilic archaea, Mo is usually replaced by tungsten (W) bound to the same unique pyranopterin (Wco), thus forming tungstoproteins [5, 6]. It has been reported that W is needed for enzymes within the aldehyde:ferredoxin oxidoreductase (AOR) family and some other enzymes belonging to molybdoprotein families in certain prokaryotes [7–10]. Although molybdoproteins and tungstoproteins appeared to have a preference for either of the two metals in many cases [8–10], the presence or exchange of both metals in certain enzymes has also been reported [11]. So far it is very difficult to distinguish the utilization of Mo and W in the majority of molybdoproteins due to similar physical-chemical and functional properties between them. Therefore, the term Moco generally refers to the utilization of both metals if not specified.

In all organisms that utilize Moco, Mo uptake and the Moco biosynthetic pathway are essential for Moco utilization [12, 13]. Three molybdate/tungstate ATP binding-cassette (ABC) transport systems (ModABC, WtpABC and W-specific TupABC) have been reported in prokaryotes [14–16]. In eukaryotes, the first identified molybdate transporter is MOT1, which is present in plant-type eukaryotic organisms such as land plants and green algae [17, 18]. A second type of molybdate transporter, MOT2, has recently been identified in algae and animals including humans, which might be involved in the uptake of Mo from extracellular space [19, 20]. Biosynthesis of the basic form of Moco involves three steps which are quite similar between prokaryotes and eukaryotes: (1) formation of a cyclopyranoperin monophosphate (cPMP) intermediate from GTP; (2) transformation of cPMP into mature pyranopterin; and (3) insertion of Mo into molybdopterin to form Moco [1, 2, 12, 13]. In bacteria such as *Escherichia coli*, six loci (*moa*–*mog*) comprising 16 genes have been implicated in this process [21]. In eukaryotes, at least six gene products (CNX1–3 and CNX5–7 as named in plants) are involved in the biosynthesis of Moco, which are homologous to their counterparts in bacteria [2, 22]. Additional proteins such as Moco sulfurase, Moco carrier protein and Moco-binding proteins might also be involved in cellular distribution of Moco [23–25].

Almost all molybdoenzymes catalyze diverse redox reactions in the global carbon, nitrogen, and sulfur cycles [12, 26]. More than 50 molybdoenzymes have been characterized in different organisms (mostly bacteria), which could be divided into five families: sulfite oxidase (SO), xanthine oxidase (XO), dimethylsulfoxide reductase (DMSOR), AOR (W-specific) and MOSC(Moco sulfurase C-terminal domain)-containing protein (including YcbX and YiiM in bacteria and mitochondrial amidoxime reducing component (mARC) in eukaryotes) [12, 13, 26–29]. Recently it has been suggested that the MOSC-containing proteins should be considered as new members of the SO family since structures for the Mo-binding domains of these proteins are similar to those of SO and nitrate reductase [21, 26]. However, such an alternative classification approach is still controversial as the MOSC-containing proteins lack significant sequence similarity to members of the SO family [30].

The majority of previous studies have primarily focused on the functions of molybdoenzymes. Although several comparative analyses of Mo utilization in a limited number of sequenced bacterial and eukaryotic genomes have revealed a wide distribution of organisms that use Mo [31–33], it is still unclear how this transition metal is used by different organisms and whether evolution of the Mo utilization trait (either Moco biosynthesis system or molybdoproteins) could be influenced by various ecological conditions. With recent advances in high-throughput sequencing techniques, genomes of a large number of prokaryotic and eukaryotic organisms have been decoded. These data provide new opportunities to explore the evolutionary dynamics of Mo utilization.

In this study, we performed a comprehensive analysis of the occurrence and evolution of Mo utilization in approximately 5900 sequenced organisms from archaea, bacteria and eukaryotes, which generated the largest and most detailed atlas of Mo utilization in all three domains of life. Distributions of Mo/W transporters, Moco biosynthetic pathway and molybdoprotein families were identified. A variety of fusion forms of molybdoproteins were detected, which highlight functional links between them and some other proteins. Further investigation of the whole set of molybdoproteins (molybdoproteome) of each Mo-utilizing organism revealed the presence of new molybdoprotein-rich organisms. Finally, the relationship between Mo utilization and environmental factors showed that the Mo utilization trait may favor specific environmental conditions. In general, these data advance our understanding of how Mo is used by a wide range of organisms and how the distribution and functions of molybdoenzymes have been influenced by evolutionary processes.

## Results

### Occurrence of the Mo utilization trait in prokaryotes and eukaryotes

**Fig. 1:**
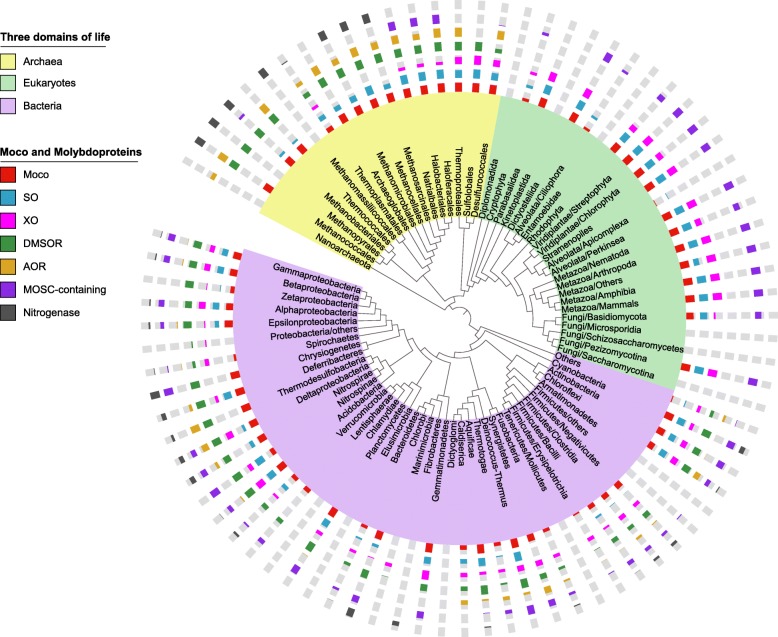
Distribution of the Moco biosynthetic pathway and molybdoenzyme families in bacteria, archaea and eukaryotes. The phylogenetic tree is simplified to only show major taxa and branches of bacteria (light purple background), archaea (light yellow background) and eukaryotes (light green background). The seven tracks (circles) around the tree (from inside to outside) represent the distribution patterns of Moco (Moco biosynthetic pathway), SO (sulfite oxidase), XO (xanthine oxidase), DMOSR (dimethylsulfoxide reductase), AOR (aldehyde:ferredoxin oxidoreductase), MOSC-containing protein and nitrogenase families, respectively. The length of the colored section of each column represents the percentage of organisms that possess either Moco biosynthetic pathway or the corresponding molybdoenzyme families among all sequenced organisms in this branch

Previously, we have analyzed the distribution of the Mo utilization trait in several hundred organisms [31, 32]. In this study, we expanded such analysis to a much broader set of sequenced organisms from both prokaryotes and eukaryotes, whose number was eight times larger than previous studies. Our data demonstrated the largest Mo utilization map in all three domains of life thus far. An overall view of Mo utilization in different taxa is shown in Fig. 1 (details are shown in Additional file 1: Table S1-S3).

The majority of sequenced organisms in each kingdom use Mo (Table 1). In bacteria, a total of 3683 (66.8%) organisms were found to utilize Mo. Except for the phyla containing very few sequenced genomes (≤5), the Mo utilization trait was detected in almost all examined subdivisions (Fig. 1). In particular, all sequenced organisms in Aquificae, Chlorobi, Cyanobacteria, Deferribacteres, Deinococcus-Thermus, Nitrospirae, Planctomycetes and Thermodesulfobacteria, as well as the majority of Acidobacteria (95.5%), Deltaproteobacteria (90.5%), Epsilonproteobacteria (89.5%), Gammaproteobacteria (87.5%) and many other bacterial taxa utilize Mo. In contrast, a small number of clades such as Chlamydiae, Tenericutes/Mollicutes and Marinimicrobia were found to lack the Mo utilization trait. A much wider occurrence of Mo utilization was observed in archaea (249 out of 256 organisms, 97.3%). Only seven sequenced organisms (including all sequenced Nanoarchaeota) lacked this trait, suggesting that Mo utilization is an essential trait for nearly all species in this domain of life. In addition, most nitrogenase-containing organisms possess the Moco utilization trait (87.7% and 90.4% for bacteria and archaea, respectively), implying that the occurrence of nitrogenase is highly related to that of the Moco biosynthetic pathway, probably due to the already-existing Mo transport systems.

**Table 1:** Distribution of Mo utilization trait in archaea, bacteria and eukaryotes

Kingdom	All sequenced organisms	Mo-utilizing organisms	Organisms only containing Moco utilization trait	Organisms only containing nitrogenase	Both^a^
Archaea	256	249	176	7	66
Bacteria	5387	3683	3120	69	494
Eukaryotes	250	176	176	–	–

^a^Organisms containing both the Moco utilization trait and nitrogenase

In eukaryotes, a total of 176 (70.4%) organisms were found to have the Mo utilization trait, especially all organisms in Dictyosteliida, Fungi/Pezizomycotina, Stramenopiles, Viridiplantae and Metazoa (Fig. 1). However, most alveolata (such as Ciliophora and Apicomplexa), yeasts (Saccharomycotina and Schizosaccharomycetes) and a number of parasitic protists responsible for serious diseases in humans and other animals (such as Diplomonadida, Entamoebidae and Kinetoplastida) appeared to have lost the ability to utilize Mo.

Almost all Mo-utilizing organisms possess both the Moco biosynthetic pathway and known molybdoproteins, indicating a good correspondence between them. Some prokaryotic organisms were found to contain only part of the Moco utilization trait (Table 2). The majority of these organisms contain highly similar homologs of different Moco-dependent molybdoprotein families but lack a complete Moco biosynthetic pathway (orphan molybdoprotein-containing organisms), most of which even lack any known Mo transporters (Additional file 1: Table S1 and Table S2). Considering that many of these organisms have not been completely sequenced or assembled, the possibility that other known genes involved in Moco biosynthesis have not been sequenced could not be fully excluded. Nevertheless, the widespread occurrence of the Mo utilization trait in both prokaryotes and eukaryotes observed here is consistent with our previous assumption that Mo utilization is an ancient trait and was once common to almost all species in the three domains of life.

**Table 2:** Identification of organisms containing part of the Moco utilization trait

Kingdom	Moco (+), Moco-dependent molybdoenzyme (−)		Moco (−), Moco-dependent molybdoenzyme (+)	
	Nitrogenase (+)	Nitrogenase (−)	Nitrogenase (+)	Nitrogenase (−)
Archaea	–	–	2	3
Bacteria	1	2	13	292

### Distribution of Mo/W uptake systems

**Fig. 2:**
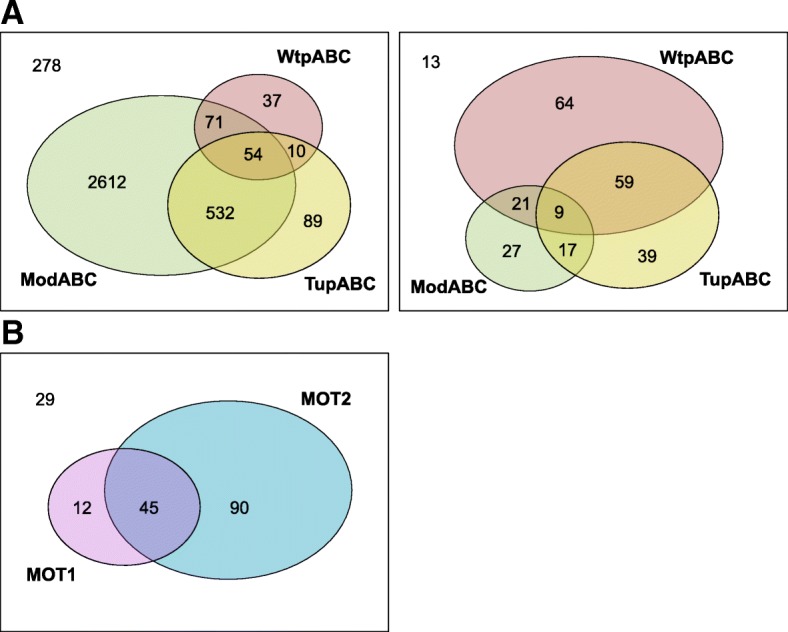
Comparison of different Mo transport systems in prokaryotes and eukaryotes. The Venn diagram is used to show the overlaps of different transporters in each kingdom. **a** Prokaryotes (left: bacteria; right: archaea): three classes of high-affinity Mo/W ABC transport systems are known: ModABC, WtpABC and TupABC; **b** Eukaryotes: two classes of Mo transporters are known: MOT1 and MOT2

We examined the occurrence of known Mo/W transporters in prokaryotes (Table 3). More than 90% Mo-utilizing organisms have at least one known Mo transport system, many of which contain two or more of them (Fig. 2a). In bacteria, ModABC was the most abundant Mo transport system, which was detected in 3269 (88.8%) Mo-utilizing organisms. The occurrence of TupABC and WtpABC was much more restricted, which accounted for 18.6% and 4.7% of all Mo-utilizing organisms, respectively. In archaea, WtpABC appeared to be the most common transporter, which was detected in 153 (61.4%) Mo-utilizing organisms, whereas ModABC had a much lower occurrence (29.7%) in this kingdom. These data are consistent with previous observation that ModABC is mainly a bacterial Mo transporter, while WtpABC functions predominantly in archaea [31]. However, TupABC was detected in 49.8% of Mo-utilizing archaea, whose occurrence is nearly two times higher than that in bacteria. It seems that TupABC has played a more important role in W uptake in archaea. We also checked the occurrence of a distant group of ModABC transporter (ModABC-like) which was previously predicted in several *Pyrobaculum* species in archaea [31]. Only seven *Pyrobaculum* species and *Thermoproteus tenax Kra 1* were found to have this subfamily (Additional file 1: Table S2), implying that it has only recently evolved in Thermoproteales.

**Table 3:** Distribution of Mo transport systems

Kingdom	Mo-utilizing organisms	ModABC	WtpABC	TupABC (W)	MOT1	MOT2	No known transporter
Archaea	249	74	153	124	–	–	13
Bacteria	3683	3269	172	685	–	–	278
Eukaryotes	176	–	–	–	57	135	29

A small number of organisms which contain the Mo utilization trait did not possess any of the known transporters (Table 3, Additional file 1: Table S4 and Table S5). Most of these organisms are distantly related, free-living organisms. This observation suggests that additional Mo/W uptake systems may exist. It is possible that molybdate/tungstate is transported by either sulfate transport system or nonspecific anion transporter in these organisms [34].

In eukaryotes, 57 (32.4% of Mo-utilizing organisms) and 135 (76.7%) eukaryotic organisms were found to possess MOT1 and MOT2, respectively, with an overlap of 45 organisms (Fig. 2b and Table 3). MOT2 was detected in almost all phyla that contain Mo-utilizing organisms while MOT1 was only present in several organisms that belong to Alveolata/Perkinsea, Cryptophyta, Fungi/Pezizomycotina, Stramenopiles, Viridiplantae/Streptophyta (land plants) and Viridiplantae/Chlorophyta (green algae) (Additional file 1: Table S6). All or almost all sequenced stramenopiles, land plants and green algae possess both transporters, implying that the two proteins are essential for Mo transport and homeostasis in these organisms. Similar to prokaryotes, a small number of Mo-utilizing organisms, especially all or almost all Mo-utilizing organisms belonging to Alveolata/Ciliophora and Metazoa/Arthropoda, lack both Mo transporters (Table 3, Additional file 1: Table S6), suggesting the presence of a currently unknown Mo transport system encoded in their genomes.

### Distribution of molybdoproteins and molybdoproteomes

**Fig. 3:**
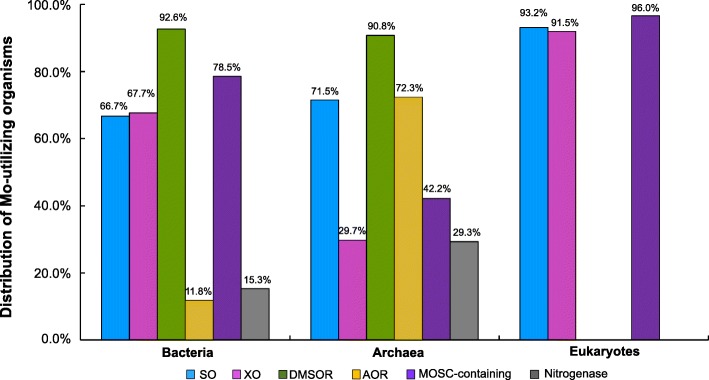
Distribution of molybdoprotein families in Mo-utilizing organisms. SO, sulfite oxidase; XO, xanthine oxidase; DMSOR, dimethylsulfoxide reductase, AOR, aldehyde:ferredoxin oxidoreductase

We analyzed the occurrence of all known molybdoprotein families (including nitrogenase) in currently sequenced genomes of both prokaryotes and eukaryotes (Fig. 3). Our results greatly extend previous analysis of molybdoprotein families in a limited number of organisms [33].

In bacteria, DMSOR was the most abundant molybdoprotein family whose members (mainly represented by formate dehydrogenase (FDH), dissimilatory nitrate reductase and DMSOR) were present in 92.6% Mo-utilizing organisms. Three other families, including MOSC-containing, XO (such as xanthine dehydrogenase (XDH), aldehyde oxidase (AO) and some other subfamilies) and SO (such as SO and assimilatory nitrate reductase) families, were also widespread in the majority of Mo-utilizing organisms (78.5%, 67.7% and 66.7%, respectively). In contrast, AOR and Fe-Mo-containing nitrogenase had a quite low occurrence (less than 20% of Mo-utilizing bacteria), implying that the two protein families are only needed by a limited number of organisms. In archaea, DMSOR was also the most widely used molybdoprotein family, which was detected in 90.8% of Mo-utilizing organisms. The AOR family had a much higher occurrence in archaea (72.3%), which was even higher than that of SO and MOSC-containing protein families (71.5% and 42.2%, respectively). The occurrence of the rest two families, XO and nitrogenase, was quite similar (29.7% and 29.3%, respectively), and the latter was only detected in methanogenic archaea.

It has been known that eukaryotes only have few members of three molybdoprotein families: SO (assimilatory nitrate reductase and SO), XO (XDH and AO) and MOSC-containing protein (mARC) families [26, 32]. Here, the three families were detected in almost all Mo-utilizing organisms (96.0%, 93.2%, 91.5% for mARC, SO and XO, respectively), suggesting that all of them are important for maintaining the proper function of Mo in Mo-utilizing eukaryotes.

**Fig. 4:**
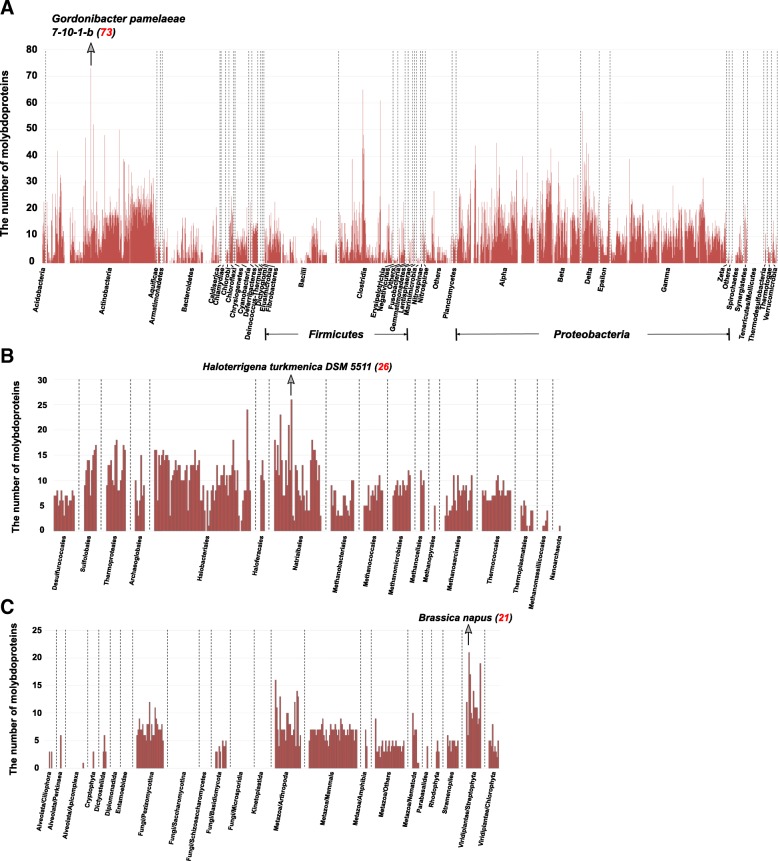
Distribution of molybdoproteomes in bacteria, archaea and eukaryotes. Organisms containing the largest molybdoproteomes in each kingdom are indicated. **a** Bacteria; **b** Archaea; **c** Eukaryotes

We further characterized the molybdoproteomes encoded in all sequenced genomes from the three domains of life (Fig. 4; details are shown in Additional file 1: Table S1-S3). In bacteria, organisms belonging to Actinobacteria and several subdivisions of Proteobacteria (such as Alpha-, Beta-, Delta- and Gamma-proteobacteria) appeared to have larger molybdoproteomes than others. A total of 309 organisms were considered as molybdoprotein-rich organisms (defined in Methods). Previously, the largest molybdoproteome was reported in a dehalorespiring bacterium, *Desulfitobacterium hafniense*, which contained at least 63 molybdoproteins (95% were members of the DMSOR family) [32]. In this study, a new bacterial species, *Gordonibacter pamelaeae 7–10-1-b* (Actinobacteria), was found to have 73 molybdoprotein genes, 69 of which encoding different members of DMOSR (Fig. 4a). This organism was isolated from the colon of a patient suffering from acute Crohn’s disease [35]. Our results suggest that DMSOR is particularly important for the living of this organism in the host.

Very few molybdoprotein-rich organisms were observed in archaea and eukaryotes. In archaea, *Haloterrigena turkmenica DSM 5511* (Natrialbales) had the largest molybdoproteome (26 molybdoproteins, Fig. 4b). In eukaryotes, land plants and arthropoda have larger molybdoproteomes than other clades. *Brassica napus* had the largest molybdoproteome (21 molybdoproteins, Fig. 4c) in this kingdom. Compared with previous results, the record of the size of molybdoproteomes in each domain of life has been renewed by this study.

### Novel domain fusions involving molybdoproteins

It has been known that gene fusion events may provide valuable information for the inference of protein interactions [36]. In this study, we identified a variety of domain fusions for each of the molybdoprotein families. The majority of these domains are known to be functionally related to certain molybdoproteins or Moco biosynthesis. For example, XDH FAD-binding subunit was found to be fused with XDH Moco-binding subunit (XO family) in hundreds of organisms in both bacteria and eukaryotes; nitrate reductase delta and gamma subunits are fused with nitrate reductase alpha subunit (DMSOR family) in some bacterial species; cysteine desulfurase has been known to serve as primary sulfur-providing protein for the biosynthesis of Moco [37].

**Fig. 5:**
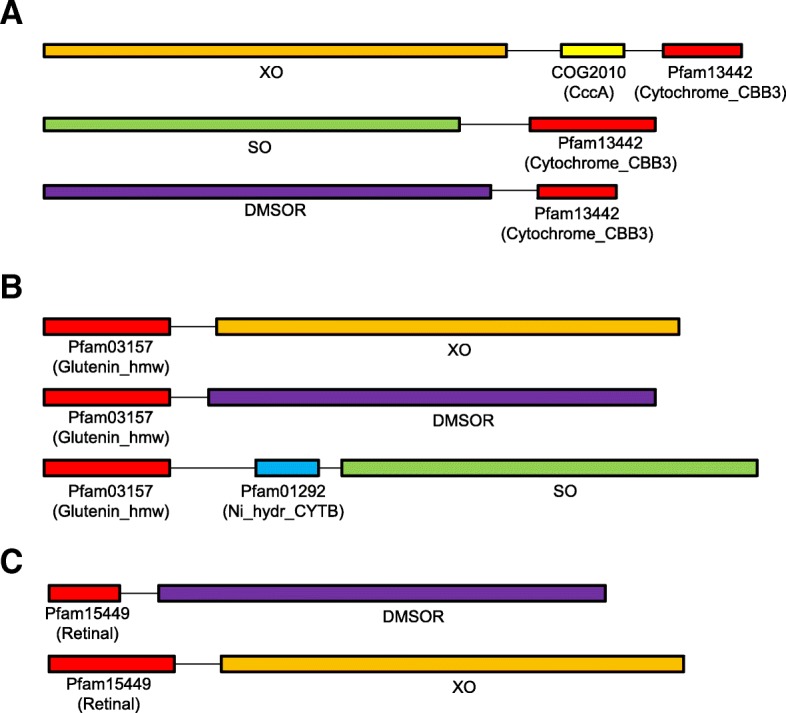
New domain fusions involving multiple molybdoprotein families. **a** pfam13442 (cytochrome C oxidase, cbb3-type, subunit III); **b** pfam03157 (high molecular weight glutenin subunit); **c** pfam15449 (retinal protein with unknown function)

We also observed several new domain fusions for DMSOR, SO and/or XO families in prokaryotes, which have not been reported to be associated with either molybdoproteins or Moco utilization (Table 4). The majority of these domains were only detected within single molybdoprotein families, implying that they are functionally related to the corresponding proteins. Interestingly, three domains, pfam13442 (cytochrome C oxidase, cbb3-type, subunit III), pfam03157 (high molecular weight glutenin subunit), and pfam15449 (retinal protein with unknown function), were found to be fused with members of multiple molybdoprotein families (Fig. 5). Homologs of cbb3-type cytochrome c oxidase (COX) subunit III were previously observed to be fused with members of the SO family when analyzing molybdoproteomes in a marine metagenomic project (manuscript submitted). In this study, we found that some other molybdoproteins, such as members of XO (also containing COG2010 (CccA, cytochrome c mono- and diheme variants)) and DMSOR, were also fused with this domain. This may suggest that certain homologs of cbb3-type COX subunit III might be involved in Moco utilization or maturation for multiple molybdoproteins in prokaryotes. Functions of the other two domains are not clear; however, fusions detected between part of them and members of different molybdoprotein families suggest that they might also be related to the general utilization of Moco.

**Table 4:** New domain fusions identified for different molybdoprotein families

Domain ID	Description	Molybdoprotein family		
		DMSOR	SO	XO
Cytochrome_CBB3 (pfam13442)	Cytochrome C oxidase, cbb3-type, subunit III	+	+	+
Glutenin_hmw (pfam03157)	High molecular weight glutenin subunit	+	+	+
Retinal (pfam15449)	Retinal protein (unknown function)	+	–	+
GltD (COG0493)	NADPH-dependent glutamate synthase beta chain or related oxidoreductase	+	–	–
YdhU (COG4117)	Thiosulfate reductase cytochrome b subunit	–	+	–
YjbI (COG1357)	Uncharacterized protein YjbI	–	+	–
GuaA1 (COG0518)	GMP synthase - Glutamine amidotransferase domain	+	–	–
Trypan_PARP (pfam05887)	Procyclic acidic repetitive protein (PARP)	+	–	–
CitB (COG2197)	DNA-binding response regulator, NarL/FixJ family	+	–	–

### Evidence of new horizontal gene transfer of the whole Mo utilization trait

Horizontal gene transfer (HGT) is an important mechanism for the spread of various biological processes in bacteria. Previously, it has been reported that some Mycobacterium species could acquire homologs of genes involved in Moco biosynthesis from Betaproteobacteria by HGT [38, 39]. In this study, we reconstructed the phylogenies for the key components of Moco biosynthetic pathway based on thousands of examined species.

**Fig. 6:**
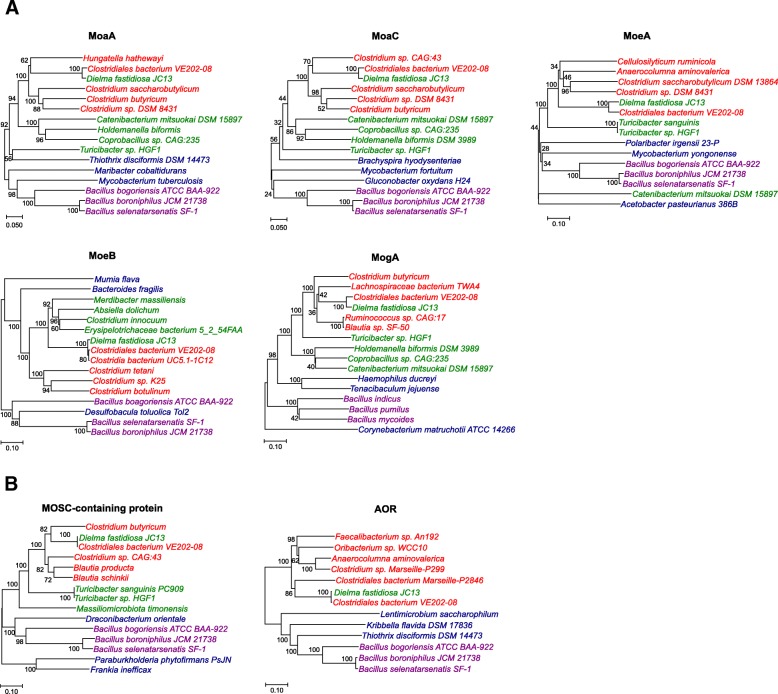
HGT of the entire Mo utilization trait. Organisms from different phyla or clades are shown in different colors. Red: Firmicutes/Clostridia, green: Firmicutes/Erysipelotrichia, purple: Firmicutes/Bacilli, blue: Others. The branch lengths and bootstrap values are also shown. **a** Moco biosynthetic pathway; **b** Molybdoproteins

An interesting observation is the clustering of *Dielma fastidiosa JC13* (Firmicutes/Erysipelotrichia) with a variety of Firmicutes/Clostridia species (especially *Clostridiales bacterium VE202–08*) in the phylogenetic trees of all key Moco biosynthesis enzymes detected in this organism (MoaA, MoaC, MoeA, MoeB and MogA, Fig. 6a). The *D. fastidiosa JC13* genome encodes two molybdoproteins (one MOSC-containing protein and one AOR), which is entirely distinct from molybdoproteins detected in other Erysipelotrichia species (only containing XO) but is quite similar to those in many Clostridia species. Phylogenetic analyses of MOSC-containing protein and AOR families revealed a similar evolutionary relationship between *D. fastidiosa JC13* and Clostridia species (Fig. 6b). In addition, almost all genes encoding the Moco biosynthetic pathway, MOSC-containing protein and AOR are located close together or arranged in an operon in the genome of *D. fastidiosa JC13* (Additional file 2: Figure S1), indicating that a very recent HGT of Mo utilization traits might have happened in this organism. Since *D. fastidiosa JC13* and *C. bacterium VE202–08* share quite similar environment (both were isolated from human faecal samples), it is likely that such an HGT event took place within certain host-associated habitat.

### Re-investigation of the relationship between Mo utilization and environmental factors

Previous studies suggested that host-associated lifestyle often led to the loss of Moco utilization trait and that different molybdoenzymes were subject to independent and dynamic evolutionary processes [31, 32]. Considering an insufficient number of genomes examined at that time, it is necessary to re-investigate such relationship using much more sequenced genomes belonging to a wider range of clades. In this study, we collected the information about living conditions for all sequenced prokaryotic organisms, and analyzed the contribution of each of these factors to Mo utilization. Considering that almost all archaea use Mo and that the majority of sequenced archaea were isolated from aquatic and anaerobic conditions, bias could be introduced when analyzing the relationship between environmental factors and Mo utilization. Therefore, we only analyzed such relationship in bacteria, which should be more reasonable to demonstrate a general evolutionary trend of Mo utilization in this kingdom.

**Fig. 7:**
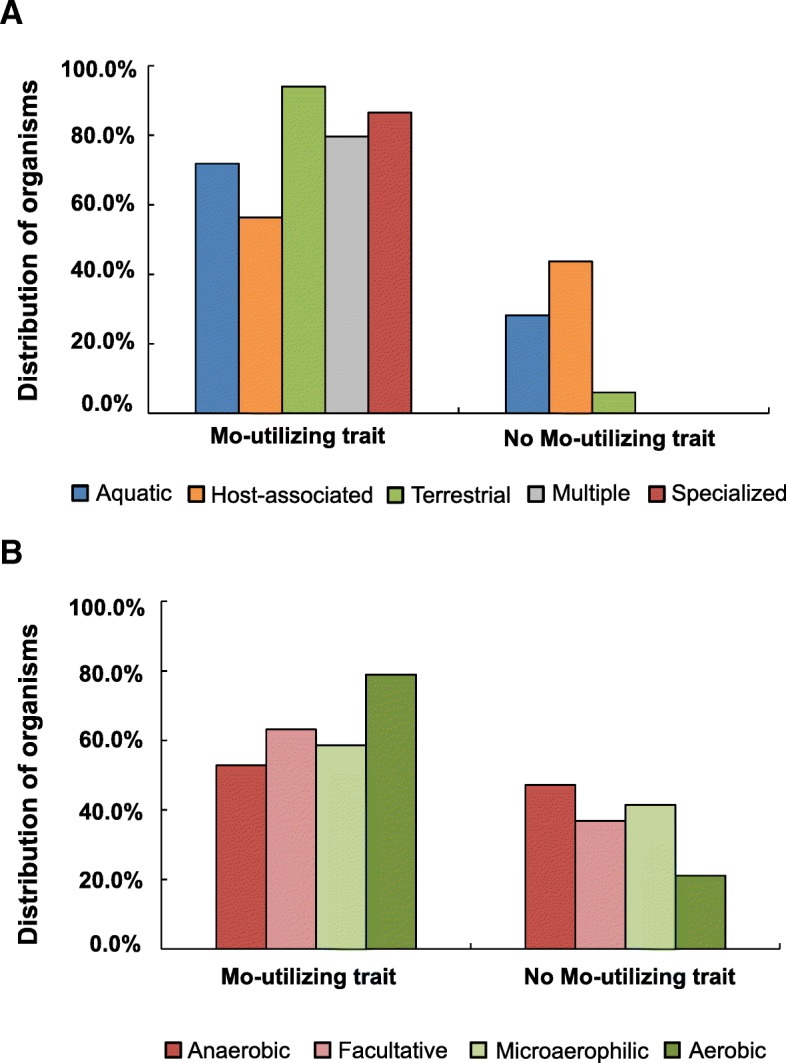
Relationship between the Mo utilization trait and several environmental factors in prokaryotes. **a** Habitat: five types were analyzed, including host-associated, aquatic, terrestrial, multiple and specialized; **b** Oxygen requirement: four types were analyzed, including anaerobic, facultative, microaerophilic and aerobic

First, habitat analysis revealed that the Mo utilization trait was most actively used by terrestrial organisms (94.05% of all sequenced terrestrial bacteria); however, only 56.30% host-associated organisms contain this trait (Fig. 7a). This is generally consistent with previous hypothesis that the use of Mo might be inhibited in host-associated condition [31, 32]. Further analysis of Mo/W transport systems showed that ModABC was most frequently used by terrestrial organisms, but TupABC might play a more important role in aquatic environments (Additional file 2: Figure S2A). With regard to molybdoproteins, except for AOR which was mainly detected in aquatic organisms, other molybdoprotein families showed a similar trend as the Mo utilization trait (Additional file 2: Figure S2A). Second, the highest proportion of Mo-utilizing organisms was found in aerobic organisms (78.92%) while approximately half of anaerobic bacteria do not use Mo, implying that oxygen has played an important role in the general evolution of Mo utilization (Fig. 7b). Analysis of Mo transporters suggested that ModABC favored an aerobic condition. In contrast, organisms possessing the other two transporters showed a wider distribution in anaerobic environments (Additional file 2: Figure S2B). As for molybdoproteins, organisms possessing AOR or nitrogenase proteins preferred anaerobic environments while organisms containing SO, XO, DMSOR or MOSC-containing proteins favored aerobic conditions (Additional file 2: Figure S2B). In addition, larger molybdoproteomes were mainly found in aerobic organisms. Other factors (such as optimal temperature, optimal pH) had no significant effect on the evolution of Mo utilization. Therefore, our results clearly support previous observations and suggest that free-living, terrestrial and aerobic conditions may help to maintain the Mo utilization trait. A future challenge would be to discover additional evolutionary patterns of Mo utilization and molybdoproteomes in the three domains of life.

## Discussion

It has been known for a long time that Mo plays a very important role in a variety of organisms including bacteria, archaea, fungi, algae, plants and animals where it forms part of the active sites of a wide range of metalloenzymes [20–22, 40]. Much effort has been devoted to characterizing the mechanism of Moco biosynthesis and the functions of molybdoenzymes [1–3, 12–14, 41]; however, evolution of the Mo utilization trait remains largely unclear. In this study, we examined the occurrence and evolution of Mo (including W) utilization at the levels of Moco biosynthetic pathway, Mo/W transporters and molybdoproteins in both prokaryotes and eukaryotes based on a significantly increased number of sequenced genomes. To our knowledge, these data represent the largest and most comprehensive view of Mo utilization in all three domains of life.

Comparative analysis of Mo utilization trait revealed that this trace element could be widely utilized by almost all prokaryotic and eukaryotic phyla examined in this study, which further confirms our previous hypothesis that Mo could have been used by essentially all organisms, whereas parallel loss of this trait occurred in a limited number of clades [31, 32]. Interestingly, we identified several hundred organisms that contain part of the Mo utilization trait, especially those containing orphan molybdoproteins (mostly DMSOR and XO families; Additional file 1: Tables S1 and S2) with the absence of a complete Moco biosynthetic pathway. Although the possibilities that some of these genes are pseudogenes or that other known genes involved in Moco biosynthesis have not been sequenced could not be excluded, our findings may imply the presence of either new Moco biosynthetic components or Mo-independent form of these proteins. It has been reported that some metalloproteins either include both metal-dependent and metal-independent forms or have evolved to use other metals in different organisms [42]. In most cases, metal-independent forms of metalloproteins have lost specific residues or lacked a complete metal-binding motif. In spite that the Moco-binding ligands in most molybdoproteins are not clear yet, we tried to search for specific residues that might be associated with Mo binding in those protein families (say, exclusively present or absent in orphan molybdoprotein sequences). Unfortunately, no significant sequence-level features could be identified. Urgent efforts are needed to verify whether or not these orphan proteins use Mo/W.

Analyses of Mo/W transport systems and molybdoproteins provided a straightforward way to demonstrate the distribution and evolution of each of these families in Mo-utilizing organisms. In prokaryotes, ModABC and WtpABC were the most abundant Mo transporters in bacteria and archaea, respectively. The W-specific transport system, TupABC, was found to have a much more important role in archaea than in bacteria, implying that W is more frequently used by certain molybdoproteins in archaea (probably due to the wide occurrence of AOR proteins). Consistent with previous observations, DMSOR was still the most widespread molybdoenzyme family in both bacteria and archaea although its members are quite diverse in reaction, function and structure [32]. Interestingly, Bacteria had a higher occurrence of MOSC-containing protein (including YcbX and YiiM) than other molybdoenzyme families such as SO and XO, suggesting that this newly identified molybdoprotein family is actually essential for the majority of Mo-utilizing bacteria. On the other hand, AOR-dependent oxidation of aldehydes should be needed for most archaeal species but not for most bacteria. In eukaryotes, organisms containing MOT1 were less than half of those possessing MOT2, indicating that MOT2 is more important for Mo uptake and homeostasis in eukaryotes. Although very few molybdoenzymes belonging to SO, XO and MOSC-containing protein families have been reported in eukaryotes, essentially all Mo-utilizing organisms had members of all these three families, implying that they are all crucial for almost all eukaryotic species that use Mo. Further investigation of the molybdoproteomes has generated the largest molybdoprotein dataset so far, which revealed high diversity of the occurrence of these proteins in different taxa. Several hundred molybdoprotein-rich organisms were identified, which may have potential implications for the development of Mo-enriched bio-products. Actinobacteria and Proteobacteria had relatively larger molybdoproteomes than other prokaryotic phyla, especially a host-associated anaerobic actinobacterium *G. pamelaeae* which has the largest molybdoproteome reported to date. In eukaryotes, besides land plants that are known to have larger molybdoproteomes [32], several arthropods were also found to have larger molybdoproteomes (containing multiple members of the XO family) than many other organisms such as animals, suggesting that these molybdoproteins are important for the survival of these organisms.

As mentioned above, it is very difficult to understand how each of the two transition metals, Mo and W, is used by individual enzymes in most organisms. Recent theoretical and experimental advances on tungstoproteins have shed light on W utilization in specific organisms [7–11]. The currently known tungstoproteins include nearly all enzymes of the AOR family and certain enzymes of the DMSOR family, such as FDH and acetylene hydratase (ACH) from several obligately anaerobic bacteria [11, 43], and formylmethanofuran dehydrogenase (FWD) from certain methanogenic archaea [44]. Although no clear criteria (such as metal-specific motifs) have been developed to distinguish their utilization in the majority of Mo/W-utilizing organisms, we tried to predict tungstoproteins from molybdoprotein families based on similar living conditions of these organisms, which may provide a first glance at W utilization in prokaryotes. The following proteins were considered as possible tungstoproteins: (i) members of AOR; (ii) FDH and ACH orthologs in strictly anaerobic bacteria; (iii) FWD orthologs in methanogenic archaea. We found that W-containing DMSOR (represented by W-containing FDH and ACH) were present in 71.8% and 56.8% W-utilizing bacteria (verified by the presence of both the Moco biosynthetic pathway and at least one possible tungstoprotein), respectively (Additional file 2: Figure S3). However, in archaea, AOR was much more frequently used than W-containing DMSOR which mainly exists in methanogens in the form of FWD (88.7% vs. 32.0%). In addition, we found that the majority of molybdoproteins detected in *G. pamelaeae* (having the largest molybdoproteome) are ACH orthologs which were predicted as tungstoproteins (Additional file 2: Figure S4). Further studies are needed to verify whether these predicted tungstoproteins contain W.

A significant contribution of this work is to identify several new evolutionary events for Mo utilization, including domain fusions of different molybdoproteins and HGT of the entire Mo utilization trait. Except for domains that are previously known to be functionally linked to molybdoproteins or Moco biosynthesis, several new domains were also suggested to be associated with certain molybdoproteins or Moco utilization, especially pfam13442, pfam03157 and pfam15449 which were detected to be fused with members of multiple molybdoprotein families. These findings would not only provide clues for further understanding the complete process of Moco biosynthesis, but also offer new insights into the potential functions of additional proteins and their relationship with Mo utilization. On the other hand, HGT of the entire Mo utilization trait is very difficult because biosynthesis of Moco is a complex process which consists of a number of components. Here, for the first time, we observed HGT event for the co-transfer of both the Moco biosynthetic pathway and molybdoproteins, which may help to explore the evolutionary trends of Mo utilization in bacteria.

Finally, we re-examined the effect of several environmental factors on Mo utilization in bacteria, which suggests new evolutionary features of the Mo utilization trait. First, terrestrial organisms appeared to have a more active Mo utilization when compared with organisms living in other habitats, especially host-associated lifestyle which was previously thought to be correlated with the loss of the ability to use Mo [31]. Regarding individual proteins, ModABC and all molybdoprotein families (except AOR and nitrogenase) showed a quite similar trend as that of the Mo utilization trait, while TupABC and AOR might play a relatively more important role in aquatic bacteria. With regard to oxygen requirement, it is obvious that oxygen can generally promote Mo utilization in bacteria. ModABC and the majority of molybdoprotein families were more frequently detected in aerobic bacteria. In contrast, WtpABC, TupABC, AOR and nitrogenase showed a wider distribution in anaerobic organisms. In the future, it would be important to identify additional factors that may influence Mo utilization.

## Conclusions

In this study, we conducted a comprehensive comparative genomic analysis of Mo utilization. We extended previous small-scale analyses to nearly 5900 sequenced genomes in the three domains of life by analyzing the occurrence of all known Mo/W transporters, Moco biosynthetic pathway and molybdoproteins. Our data generated the largest map of Mo utilization in nature, which revealed a complex and dynamic evolutionary history for the Mo utilization trait. More importantly, we identified a variety of new domain fusions for different molybdoprotein families, suggesting the presence of new proteins that are functionally linked to either molybdoproteins or Moco biosynthesis. Phylogenetic analyses of key components of the Moco biosynthetic pathway and molybdoproteins indicated HGT for the complete transfer of the whole Mo utilization trait. Finally, we analyzed the relationship between different environmental conditions and the Mo utilization trait and found new interactions between ecological environments and Mo utilization. Our work will contribute to better understanding the general features of utilization and evolution of Mo in both prokaryotes and eukaryotes.  

## Methods

### Genomic sequences and other resources

All sequenced genomes were retrieved from the National Center for Biotechnology Information (NCBI). A total of 5893 organisms (5387 bacterial, 256 archaeal and 250 eukaryotic genomes) were analyzed (as of April 2016). Environmental information (such as habitat, oxygen requirement, and optimal growth temperature) for each organism was acquired from NCBI, Genomes Online Database (GOLD) [45] and the Integrated Microbial Genomes project database of the Joint Genome Institute (JGI-IMG) [46].

### Identification of Mo transporters, Moco biosynthesis components and molybdoproteins

We used several representative sequences of Mo/W transporters, Moco biosynthesis components and molybdoproteins as seeds to search for homologs in genomic sequences. A list of known Mo transporter systems, Moco biosynthesis components and molybdoprotein families is shown in Additional file 1: Table S7.

In prokaryotes, products of *moa* (*moaA*-*moaE*), *mod* (*modABC*) and *moe* (*moeA* and *moeB*) operons as well as *mobA* and *mogA* genes from *E. coli* [21], WtpABC from *Pyrococcus furiosus* [16], TupABC from *Eubacterium acidaminophilum* [15] were used to identify a primary set of homologous sequences via TBLASTN with a cut-off e-value of 0.01 and the alignment coverage of at least 20%. Repetitive TBLASTN searches were then performed within each clade (phylum or class) to identify additional homologous sequences using selected sequences from the primary data set. Orthologous proteins were then defined using the conserved domain databases (such as Pfam [47] and CDD [48]) and bidirectional best hits [49]. Additional analyses (such as gene neighborhood and phylogenetic analyses) were also used to help identify orthologs if needed. The occurrence of the Moco biosynthetic pathway was confirmed by the presence of most of the key genes involved in Moco biosynthesis and that of at least one gene in each of the three key steps (*moaA* and *moaC* for step 1; *moaD*, *moaE* and *moeB* for step 2; *moeA* and *mogA* for step 3). Members of known molybdoproteins (including nitrogenase) were identified using a similar strategy. Known Mo-independent proteins that are homologous to molybdoproteins, such as NADH-quinone oxidoreductase chain G (NuoG, similar to DMSOR) and nitrogenase Fe-Mo cofactor biosynthesis protein NifE (similar to nitrogenase), have been carefully excluded.

In eukaryotes, we used *Arabidopsis thaliana* MOT1 [18] and *Chlamydomonas reinhardtii* MOT2 [19] as queries to search for molybdate transporters, and *A. thaliana* Cnx1–3 and Cnx5–7 [12] to identify the pathway of Moco biosynthesis in sequenced organisms. Human proteins belonging to SO, XO and mARC families were used to detect homologous proteins in eukaryotes.

The Mo utilization trait was finally verified by the requirement for (i) the presence of the Moco utilization trait (including the Moco biosynthetic pathway and at least one Moco-dependent molybodoenzyme), or (ii) the presence of nitrogenase. An organism rich in molybdoproteins was then defined if it contains more than 20 molybdoprotein genes.

Distributions of the Moco biosynthetic pathway and molybdoproteins in different taxa of prokaryotes and eukaryotes were illustrated by using the online Interactive Tree Of Life (iTOL) [50] tool based on a dramatically expanded version of the tree of life that was developed very recently [51].

### Multiple sequence alignment and phylogenetic analysis

Multiple sequence alignment was performed using CLUSTALW [52] with default parameters. Ambiguous alignments in highly variable or gap-rich regions were excluded. Phylogenetic trees were reconstructed by MEGA (Molecular Evolutionary Genetics Analysis) software (version 7.0) [53] using neighbor-joining method, and were further evaluated by MrBayes (Bayesian estimation of phylogeny) tool [54]. Finally, the vector graphics editor Inkscape software (version 0.91) [55] was used to refine the fonts and colors of the phylogenetic trees.

## Additional files

Additional file 1: **Table S1.** Occurrence of the Mo utilization trait in bacteria. **Table S2.** Occurrence of the Mo utilization trait in archaea. **Table S3.** Occurrence of the Mo utilization trait in eukaryotes. **Table S4.** Distribution of Mo transporters in Mo-utilizing bacteria. **Table S5.** Distribution of Mo transporters in Mo-utilizing archaea. **Table S6.** Distribution of Mo transporters in Mo-utilizing eukaryotes. **Table S7.** List of Mo transporters, Moco biosynthesis components and molybdoproteins. (XLSX 488 kb)

Additional file 2: **Figure S1.** Genomic content of genes encoding the Moco biosynthetic pathway, MOSC-containing protein and AOR in *D. fastidiosa JC13*. **Figure S2.** Relationship between Mo/W transport systems, molybdoproteins and environmental factors in bacteria. **Figure S3.** Distribution of predicted tungstoprotein families. **Figure S4.** Phylogenetic analysis of ACH proteins in *Gordonibacter pamelaeae 7–10-1-b. (PDF 444 kb)*

Additional file 3: This file contains all molybdoprotein sequences analyzed in this study. (TXT 32763 kb)

## Abbreviations

ABC: ATP binding-cassette
ACH: acetylene hydratase
AO: aldehyde oxidase
AOR: aldehyde:ferredoxin oxidoreductase
COX: cytochrome c oxidase
cPMP: cyclopyranoperin monophosphate
DMSOR: dimethylsulfoxide reductase
FDH: formate dehydrogenase
FWD: ormylmethanofuran dehydrogenase
GOLD: Genomes Online Database
HGT: horizontal gene transfer
iTOL: online Interactive Tree Of Life
JGI-IMG: the Integrated Microbial Genomes project database of the Joint Genome Institute
mARC: mitochondrial amidoxime reducing component
MEGA: Molecular Evolutionary Genetics Analysis
Mo: molybdenum
Moco: molybdenum cofactor
MOSC: Moco sulfurase C-terminal domain
NCBI: National Center for Biotechnology Information
NuoG: NADH-quinone oxidoreductase chain G
SO: sulfite oxidase
W: tungsten
XDH: xanthine dehydrogenase
XO: xanthine oxidase
