# Supplementary material for: Comparative genomics of molybdenum utilization in prokaryotes and eukaryotes
Source: BMC Genomics. 2018 Sep 19;19:691. doi: 10.1186/s12864-018-5068-0 (PMC6147048; doi:10.1186/s12864-018-5068-0)
Supplement: Supplementary file 2 — Figure S1. Genomic content of genes encoding the Moco biosynthetic pathway, MOSC-containing protein and AOR in D. fastidiosa JC13. Figure S2. Relationship between Mo/W transport systems, molybdoproteins and environmental factors in bacteria. Figure S3. Distribution of predicted tungstoprotein families. Figure S4. Phylogenetic analysis of ACH proteins in Gordonibacter pamelaeae 7–10-1-b. (PDF 444 kb) [file 12864_2018_5068_MOESM2_ESM.pdf]

Supplementary figures

Figure S1. Genomic content of genes encoding the Moco biosynthetic pathway, MOSC-containing protein and AOR in *D. fastidiosa JC13*. The genes encoding the Moco biosynthetic pathway, MOSC-containing protein and AOR are shown in blue, red and yellow, respectively. Coding direction is also indicated.

Figure S2. Relationship between Mo/W transport systems, molybdoproteins and environmental factors in bacteria. (A) Habitat; (B) Oxygen requirement.

Figure S3. Distribution of predicted tungstoprotein families. The W-containing DMSOR family includes W-containing FDH and ACH.

Figure S4. Phylogenetic analysis of ACH proteins in *Gordonibacter pamelaeae 7-10-1-b*. Sequences of ACH proteins were selected from *G. pamelaeae 7-10-1-b* (grey) and some other representative organisms belonging to different phyla (black). Other protein branches of the DMSOR family are compressed and represented by family names, such as DMSOR (blue and bold), FDH (red and bold) and nitrate reductase (green and bold). Both bootstrap values and the measurement of distance for the branch lengths (shown by a bar) are indicated.

Figure S1

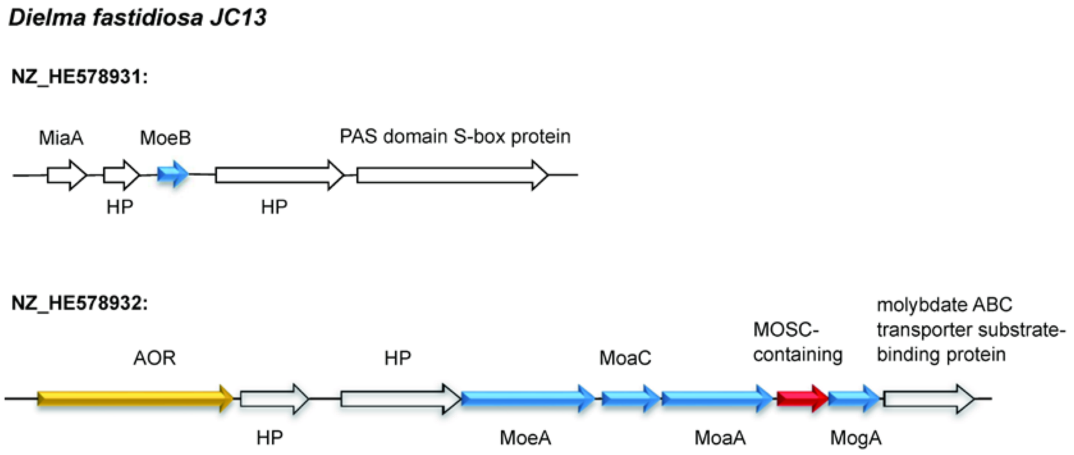
Dielma fastidiosa JC13
NZ_HE578931:
MiaA
MoeB
PAS domain S-box protein
HP
HP
NZ_HE578932:
AOR
HP
MoaC
MOSC-
containing
molybdate ABC
transporter substrate-
binding protein
HP
MoeA
MoaA
MogA

Figure S2

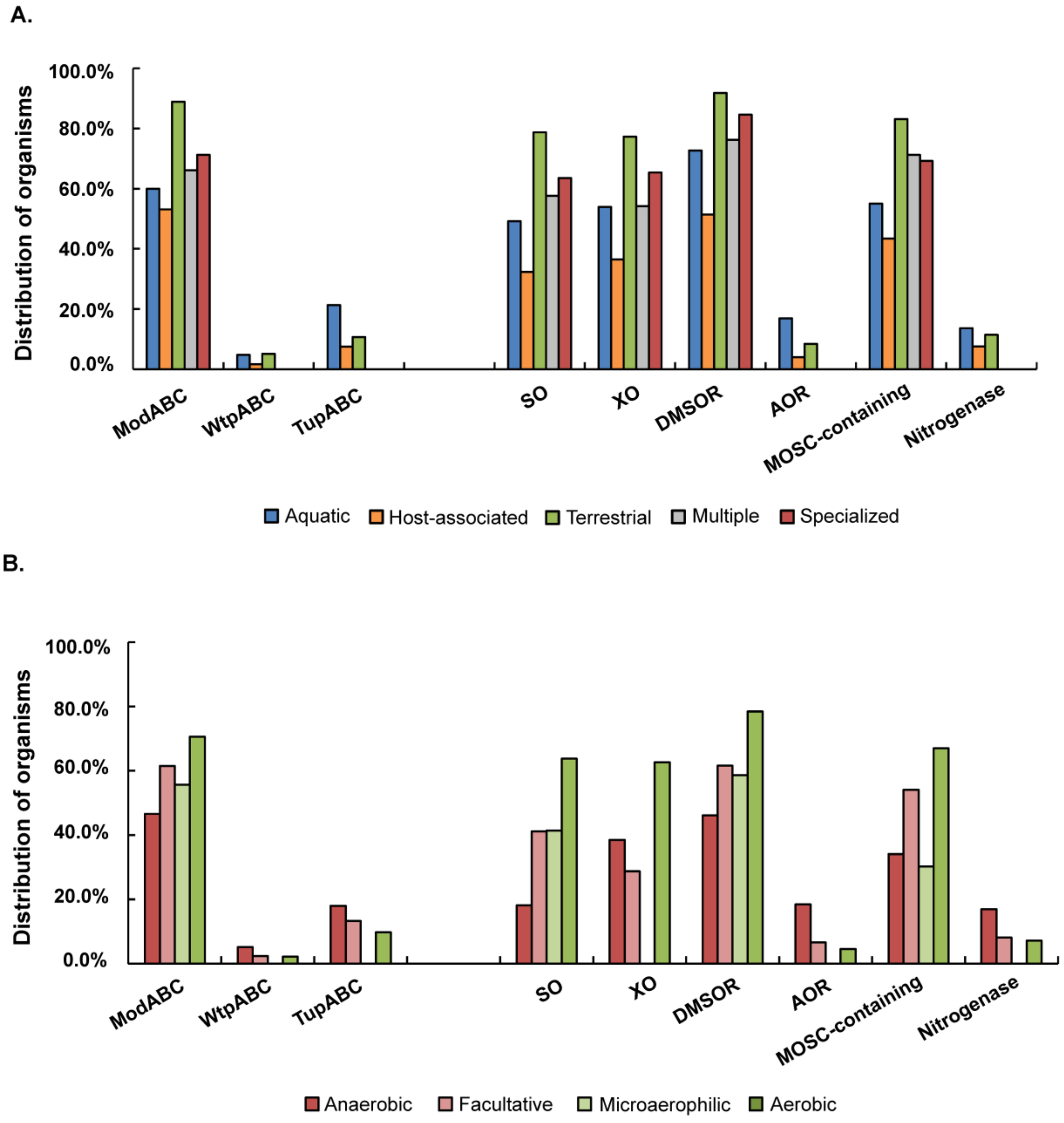
A.
Distribution of organisms
100.0%
80.0%
60.0%
40.0%
20.0%
0.0%
ModABC
WtpABC
TupABC
SO
XO
DMSOR
AOR
MOSC-containing
Nitrogenase
Aquatic
Host-associated
Terrestrial
Multiple
Specialized
B.
Distribution of organisms
100.0%
80.0%
60.0%
40.0%
20.0%
0.0%
ModABC
WtpABC
TupABC
SO
XO
DMSOR
AOR
MOSC-containing
Nitrogenase
Anaerobic
Facultative
Microaerophilic
Aerobic

Figure S3

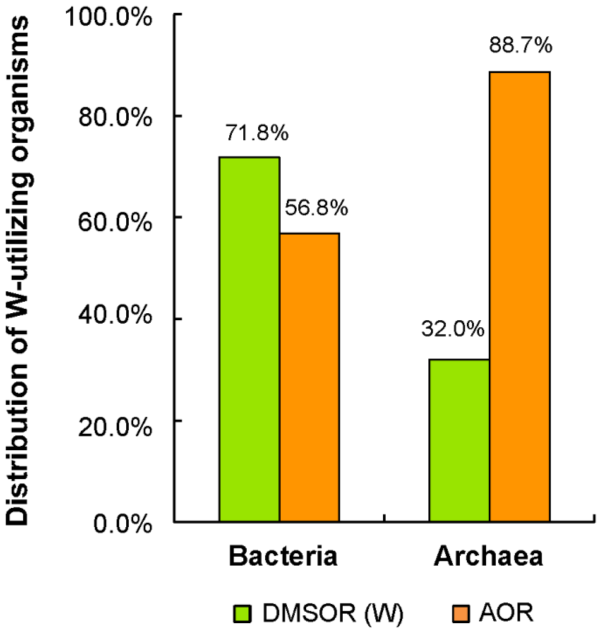
Distribution of W-utilizing organisms
100.0%
80.0%
60.0%
40.0%
20.0%
0.0%
71.8%
56.8%
32.0%
88.7%
Bacteria
Archaea
DMSOR (W)
AOR

Figure S4

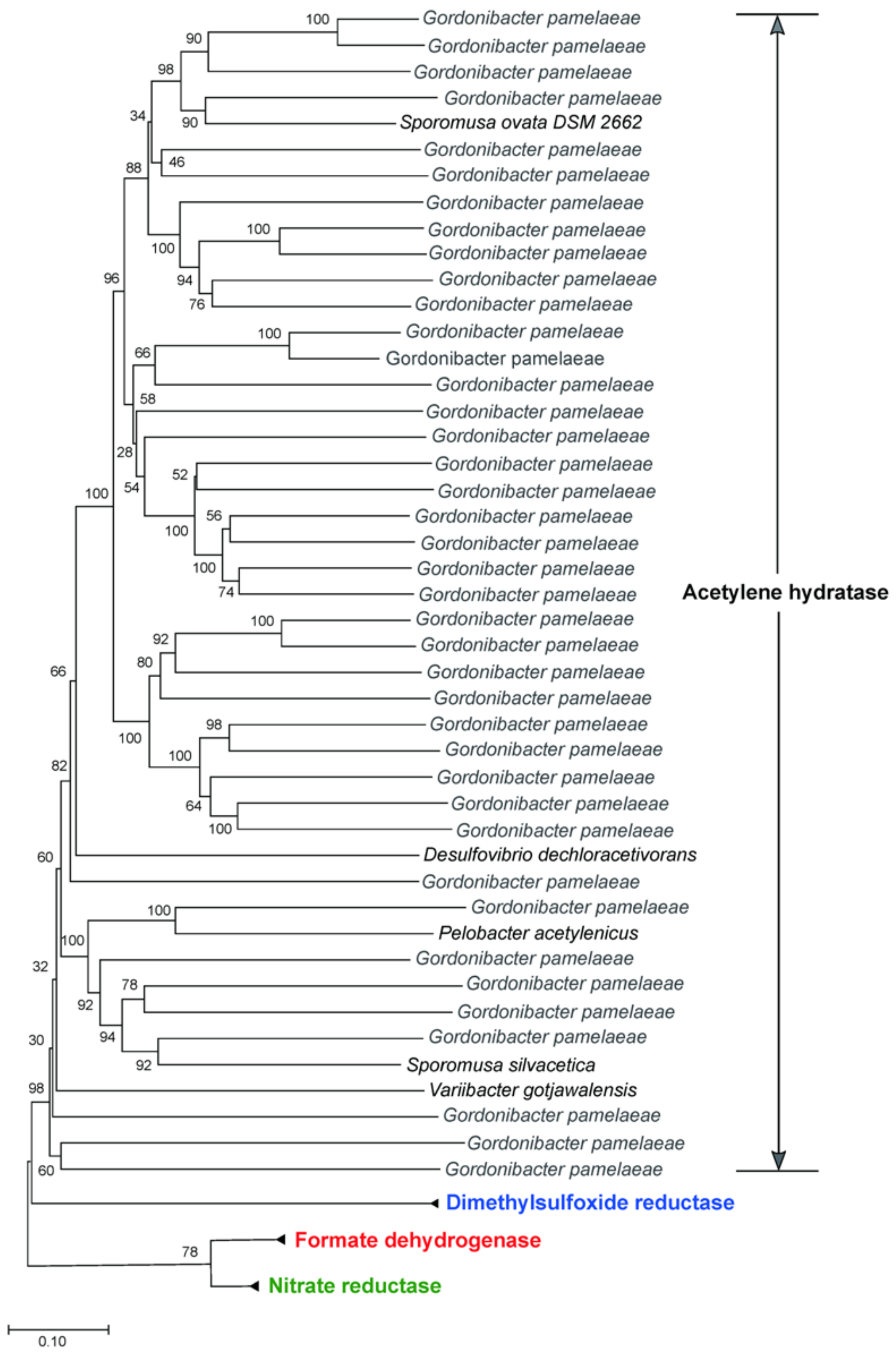

Gordonibacter pamelaeae
Sporomusa ovata DSM 2662
Desulfovibrio dechloracetivorans
Pelobacter acetylenicus
Sporomusa silvacetica
Variibacter gotjawalensis
Acetylene hydratase
Dimethylsulfoxide reductase
Formate dehydrogenase
Nitrate reductase
0.10
